# Assessing research competency development in Canadian psychiatry residency programs: A systematic review and future directions

**DOI:** 10.1177/20503121231216846

**Published:** 2023-12-12

**Authors:** Anees Bahji, Marlon Danilewitz, David Crockford, Nicholas Delva, Benjamin Rusak

**Affiliations:** 1 Department of Psychiatry, University of Calgary, Calgary, AB, Canada; 2 Department of Community Health Sciences, University of Calgary, Calgary, AB, Canada; 3 Hotchkiss Brain Institute, University of Calgary, Calgary, AB, Canada; 4 Ontario Shores Centre for Mental Health Sciences, Whitby, ON, Canada; 5 Department of Psychiatry, University of Toronto, Toronto, ON, Canada; 6 Department of Psychiatry, Queen’s University, Kingston, ON, Canada; 7 Department of Psychiatry, Dalhousie University, Halifax, NS, Canada

**Keywords:** Internship and residency, quality improvement, curriculum, psychiatry, Canada, education and training

## Abstract

**Objective:**

We aimed to conduct a systematic review to identify curricular and educational interventions to build research competency among Canadian psychiatry residents and fellows transitioning to the competency-by-design framework.

**Methods:**

The PRISMA guidelines were followed, searching five databases from their inception to February 2023 for relevant evaluation-type studies exploring research competency among psychiatry residents and fellows. We appraised thestudy’s quality using the Joanna Briggs Institute’s risk of bias tool for observational designs.

**Results:**

Overall, 36 original articles met our inclusion criteria. Surveys (*n* = 10) showed that participation in scholarly research, quality improvement, or educational projects relevant to psychiatry is needed in most residency programs. However, these vary significantly across programs; few need direct research experience for residency completion. The interventions spanned four categories: externally funded comprehensive research training programs (*n* = 5); resident research tracks (*n* = 11); workshops and seminars (*n* = 7); and specific modules (*n* = 3). Reported outcomes included overall program ratings, research output, and career trajectory. The quality of most studies was low because of the lack of controls or validated metrics for evaluating outcomes.

**Conclusions:**

While many studies have explored best practices in research curricula, the current literature does not inform competency-based models for Canadian psychiatry residency programs incorporating research training requirements. Further description is needed from Canadian psychiatric training bodies regarding appropriate curricula, milestones, and metrics for evaluating research competencies.

## Introduction

In 2001, the National Institute of Mental Health (NIMH) noted decreasing numbers of clinician scientists in psychiatry relative to other areas of medicine.^1–3^ As a result, the NIMH commissioned the Institute of Medicine to investigate the decline of research training within psychiatry residency programs in the United States.^4–6^ Consequently, research training has become an increasingly mandated component of the psychiatry residency experience in the United States to address the critical shortage of research psychiatrists.

In recent years, the landscape of psychiatric residency training in Canada has undergone a significant transformation with the adoption of competency by design (CBD). The Royal College of Physicians and Surgeons of Canada (RCPSC) has emphasized the importance of research, dedicating a CanMEDS role to scholarly development. It has specifically outlined requirements during psychiatric residency training (Appendix 1). Within the CanMEDS Scholar role, psychiatry residents must understand the scientific principles of research and scholarly inquiry; contribute to the creation and dissemination of knowledge and procedures applicable to health; describe the principles of research, scholarly inquiry, and research ethics; pose a scholarly question; conduct a systematic search for evidence; select and apply appropriate methods to address the question; and disseminate the findings of a study.

As of 1 July 2020, Canadian psychiatry residency programs shifted to CBD,^7^ which aims to prepare psychiatrists for practice by orienting training to competencies derived from the needs of society and patients.^8^ To operationalize CBD, the RCPSC has adopted “entrustable professional activities” (EPAs),^9^ which are units of professional practice entrusted to trainees demonstrating the necessary competence to execute the activity independently.^10^ Working groups in psychiatry have defined 20 EPAs across four stages of residency training, with some for specific psychiatric subspecialties.^11–34^ Although the RCPSC has described the research requirements and emphasized research as an essential component of psychiatric training,^35,36^ the only EPA related to research in the current CBD framework is early in training within the Foundations of Discipline (FoD) stage (EPA #5), which specifies that two direct observations from at least two different observers are required of the resident to “perform a critical appraisal and presenting psychiatric literature.” Key features of this EPA include: (1) “A focus on critical appraisal of literature to make appropriate clinical decisions and to encourage lifelong learning and acquisition of new knowledge and skills in the specialty”; (2) “Includes posing a clinically relevant question, performing a literature search, critically appraising the literature, and presenting in a group setting”; and (3) “Includes presentations such as grand rounds, journal club, case conference, morbidity and mortality rounds or quality improvement rounds.”

As CBD has progressed in Canadian psychiatry residency training, different program directors and competency chairs have noted issues with some of the EPAs and have been providing feedback to the Royal College. As the original cohort moves to different stages of their training, there is mounting pressure on programs to ensure that their residents achieve the current EPAs. However, the curriculum has become even more packed with the Royal College exam moving to the end of PGY4. Consequently, it may be even more difficult to address research competencies adequately. As EPAs tend to focus more on clinical skills that all generalist psychiatrists need, it is unlikely that there will be an expansion to have more research EPAs. However, other EPAs in the current CBD curriculum for psychiatry residents inherently touch on research competencies. For example, in the Core of Discipline stage, another EPA focuses on “teaching for students, residents, the public and other health care professionals.” A key feature of this EPA includes “critical appraisal of relevant literature,” but it is more applicable to presenting, where they also talk about adapting language and material to the audience’s needs and effective presentation skills.

Regrettably, the research-related EPAs provide little description or guidance on what meets the standard for individual evaluations on critical appraisal and presenting findings. Furthermore, there are no standards set for how preceptors who will evaluate this EPA must themselves have recognized skills in critical appraisal and conducting research, which could lead to unreliable assessments across sites and preceptors. There is also no description of what formal critical appraisal skills need to be demonstrated and what level of sophistication is required to meet the standard. Thus, the requirements of the EPA appear to fall short of what the RCPSC requires.^37,38^

Despite the RCPSC’s recognition of the significance of research within CBD, the implementation and evaluation of research competencies in psychiatry residency programs remain complex and multifaceted. To address this, we conducted a systematic review of educational interventions related to research for psychiatry residents and fellows. The primary goal of this review is to clarify the requirements and standards for critical appraisal and presentation skills within the CBD framework. By evaluating and synthesizing existing educational interventions, we aim to provide guidance and support to Canadian psychiatric residency training programs, helping them develop a more consistent and effective approach to meeting the CanMEDS Scholar role’s research-related expectations. This systematic review, therefore, plays a crucial role in enhancing the competency of psychiatry residents in research skills within the evolving CBD framework.

## Methods

### Protocol and registration

To provide a comprehensive synthesis of educational interventions related to research for psychiatry residents and fellows, we conducted a systematic review following the Preferred Reporting Items for Systematic Reviews and Meta-analyses (PRISMA) guidelines.^39^ Our systematic review protocol was registered with PROSPERO (CRD42021244124). We have included a checklist with the relevant PRISMA items in Appendix 2.

### Eligibility criteria

Eligibility criteria for the included studies were established based on the population-intervention-comparison-outcome-study design (PICOS) framework, which guided our selection process^40^:

1. Population: Psychiatry residents and fellows.
2. Intervention: Research-oriented educational interventions, such as courses, curricula, modules, self-assessment tools, or competency frameworks.
3. Comparator: Not required.
4. Outcomes: Psychiatry research competencies as per the RCPSC guidelines (Appendix 1).
5. Study design: We considered any evaluative study (e.g., pre-post, randomized controlled trials, surveys); we excluded commentaries, editorials, and review articles.

### Search strategy

After meeting with an experienced research librarian, we developed a systematic review protocol involving the following four databases on 16 March 2021, with an updated search on 14 February 2023, with no articles found in the next interval: EMBASE, MEDLINE, PsycINFO, and ERIC (Appendix 3). In addition, we supplemented the search by examining the reference lists of the eligible studies.

### Selection of studies

Following the removal of duplicates, one coauthor (AB) completed two independent rounds of screening (first by title and abstract and then by full-text review).

### Data extraction and management

We used Cochrane’s Covidence, a web-based systematic review manager, to extract information from each study. We contacted the study authors for data confirmation or clarification where necessary; we resolved disagreements by consensus. We pulled the following data items, aligned with the PICOS framework: study author, study design, year and location of study, sample size and demographic characteristics (e.g., age, sex, and stage of training), research aspect explored, nature and type of intervention, other outcome measures, and summary of the study’s findings.

### Assessment of risk of bias in the included studies

Recent reviews have indicated that serious concerns arise from applying scales and instruments that have not been validated to evaluate the risk of bias in observational studies’ systematic reviews.^41–44^ Therefore, we assessed the study’s quality using the Joanna Briggs Institute’s (JBI) risk of bias tool for observational studies.^45,46^ In brief, the JBI checklist appraised eight domains of study quality in observational designs, including the description of study participants, study eligibility criteria, methods for measuring and defining exposure and outcome variables, accounting for confounding factors, and choice of analytic techniques. Each of these eight domains receives a 0 or 1, with higher scores indicating better quality. Studies receiving 6 or more points were designated “High quality”; otherwise, they were “Low quality.”

### Synthesis of the findings

We undertook a descriptive synthesis as a meta-analysis was not feasible due to excessive heterogeneity in the study design and outcome reporting, including overall program ratings (e.g., participant satisfaction, confidence with a skill), research output (e.g., publications, awards, grant funding, citations), and research career trajectory (e.g., transition or matriculation to further research training, academic positions, affiliation with professional bodies). However, we identified themes from surveys on psychiatry residents that provided a context for attitudes and perceived barriers to research. In addition, we identified potential strategies that residency programs could employ to enhance research competency with their residents from intervention-type studies.

## Results

### Study selection

**Figure 1.**
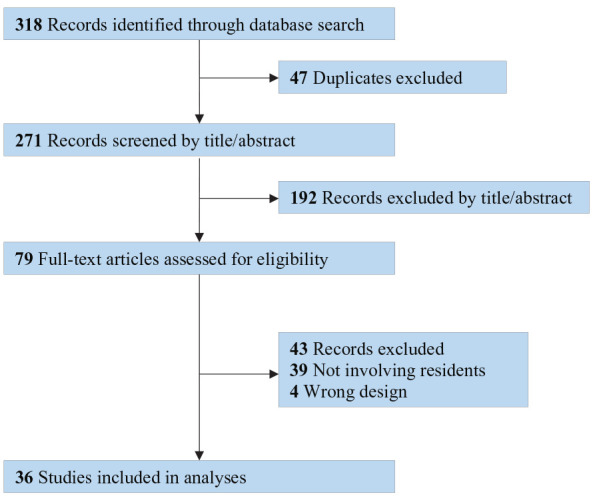
PRISMA study flow diagram.

From 318 records, 36 original articles met our inclusion criteria (Figure 1). Ten surveyed general attitudes, experiences, and reflections on research training during residency (Table 1).^47–56^ The remaining 26 studies evaluated interventions designed to enhance research competencies or skills in specific techniques (Table 2).^57–82^

**Table 1.** Characteristics of the survey studies (*n* = 10).

Study	Design and sample	Summary of findings
Hershberg et al.^47^	A national survey of Canadian psychiatric residents (*n* = 190, response rate not reported)	Residents identified research as an essential part of psychiatry, and most welcomed the idea of formal training. However, two-thirds felt that their departments were underproductive in terms of research
Strauss et al.^48^	A national survey of U.S. psychiatry residents (*n* = 115, 50% response)	Although most programs offered a research activity (e.g., journal club), minimal research resources were available to residents. The availability of research-specific teaching was highly variable, and few programs were budgeting for resident research training. Few programs require any direct research experience for residency completion
Andrews et al.^49^	A national survey of Australian psychiatry training directors (*n* = 20, 87% response)	Most directors reported offering formal research seminars, lectures, and journal clubs for residents, but only two programs formally required trainees’ participation in research
Balon and Singh^50^	A national survey of Canadian and U.S. psychiatry residency programs (*n* = 70, 59% response)	There were few research training opportunities across programs. Research didactics were relatively short, with trainees reporting a lack of education on research designs. In addition, trainees were rarely required to submit a research protocol or start a project for residency completion
Fitz-Gerald et al.^51^	A national survey of the American Association of Directors of Psychiatry Residency Training and chief residents (*n* = 399, 49% response)	The major finding of the survey pointed to a discrepancy between attitudes toward research, the need for research training requirements, and the availability of research resources. For example, 98% of respondents indicated that research training should be provided to residents, but only 32% thought that research should be required
Winter et al.^52^	A regional survey of Wessex psychiatry specialist registrars (*n* = 26, 77% response)	Most (77%) trainees developed research training plans with an identified mentor, and most (91%) received protected research time. Of the 24 trainees undertaking research, 88% felt adequately supported, 83% were interested in their research, 67% enjoyed it, and 88% gained research knowledge/skills
Silberman et al.^53^	A national survey of U.S. psychiatry senior residents from programs with class sizes of at least 10 (*n* = 127, 67% response)	Compared to residents with a low or medium research interest, residents with higher interest were more likely to be male, have graduate degrees, have previous research training experience, have greater research activity in residency, and carry lower debt
Shanmugalingam et al.^54^	A national survey of Canadian resident representatives (*n* = 15, 88% response)	Most programs implemented scholarly activity for residency completion and created guidelines on resident research requirements. However, the specific requirements varied widely, as did research resources (e.g., protected time, mentorship, funding)
Laliberté et al.^55^	A national survey of Canadian psychiatry residents (*n* = 207, 24% response)	Research interest decreased by 76% by the 5th year of residency across programs. There was no association between research track participation and improved research interests
MacMaster et al.^56^	A national survey of Canadian psychiatry residents and directors (*n* = 119, 13% response)	Nearly all (87%) rated research as essential in informing clinical practice, but only 29% reported program satisfaction. In addition, only 22% were enthusiastic or very enthusiastic about participating in research

U.S.: United States.

**Table 2.** Characteristics of the evaluation studies (*n* = 26).

Study	Design and sample	Intervention	Summary of findings
Fisher and Bender^57^	Evaluation of a NIMH-funded research training program for senior Boston University Medical Centre psychiatry residents (*n* = 19)	The authors describe a 2-year program available to senior psychiatry residents, emphasizing basic and clinical research skills training, research activity, supervision, projects, seminars, workshops, and presentations	Program participants remained active in research, published extensively, and assumed substantial teaching responsibilities in medical schools
Tuma et al.^58^	Evaluation of research mentorship workshops for select U.S. child psychiatry fellows (*n* = 34)	The authors describe three NIMH-funded 3-day workshops for fostering research among selected U.S. child psychiatry trainees. The workshops provided intensive mentorship and discussions with senior investigators in the field	Nearly 50% of trainees completed formal research training and remained actively involved in research at least one day a week. Trainee-led publications increased over time
Belmont et al.^59^	Evaluation of a model for research training for U.S. child psychiatry fellows (sample size not reported)	The model involved a 4-month research rotation and a year of biweekly research meetings. During the research rotation, residents learned research methods and participated in a long-term research project	The training encouraged residents’ future research activities. As a result, program participants remained active in research, published extensively, and assumed substantial teaching responsibilities in medical schools
Balon and Kuhn^60^	Evaluation of two innovative research training programs for Wayne State University psychiatry trainees (*n* = 9)	The authors developed, implemented, and evaluated a summer psychiatry research fellowship and a 2-year Master of Science in Psychiatry Program, which provided protected time for graduate coursework, work on a project, mentorship, and a graduate degree	Program participants remained active in research, published extensively, and assumed substantial teaching responsibilities in medical schools. They were also successful in terms of grants, awards, and citations
Halpain et al.^61^	Evaluation of a summer research training program for University of California San Diego psychiatry trainees (*n* = 123)	An annual week-long research training program followed by ongoing mentorship for selected trainees. Sessions covered scientific integrity, participant recruitment, grantsmanship, and work-life balance	Nearly 90% of attendees presented and published articles within a year of attendance. Over 50% received grant funding within 18 months. Almost 80% remained in academic positions
Gilbert et al.^62^	Evaluation of a research mentorship track for psychiatry residents at the University of Pittsburgh (*n* = 33)	The university developed a research track for its residents to address the critical need for training physician-scientists in psychiatry, including protected time and mentorship from faculty members	Trainees received 50%–75% protected research time. As a result, nearly 50% became research postdocs or full-time grant-funded researchers in academic positions
Kunik et al.^63^	Evaluation of a regional research training program for psychiatry residents at the South-Central Mental Illness Research, Education, and Clinical Center (*n* = 26)	A low-cost, innovative regional program to train psychiatry residents in research and attract them to academic careers, providing mentorship and exposure to seasoned researchers, didactic coursework, and a stipend to cover educational expenses	Program highlights included improved research enthusiasm, the increased representation of minority groups, six publications, 28 academic presentations, and 50% retention in academic research careers
Löwe et al.^64^	Controlled evaluation of a 1-year clinical research training for German residents (*n* = 15); controls were 22 residents not participating in the training program	The training program included a weekly class in clinical research methods, a research project, and mentorship. Outcomes included methodological research knowledge (multiple-choice progress test), self-assessed research competence, progress on publications and grant applications, and quantitative-qualitative program evaluations	Methodological knowledge, self-assessed research competence, and publication activity improved significantly more in the intervention group compared to the control group. The intervention subjects evaluated the training program as valuable for becoming independent researchers
Roane et al.^65^	Evaluation of the PART program for Beth Israel Medical Centre psychiatry residents (*n* = 89)	The PART program addressed the shortage of research psychiatrists by providing funding, protected research time, and mentorship to residents using an experiential research model	Following program inception, scholarly activities increased over a 5-year observation period (e.g., 32 publications, 56 presentations, and NIMH awards)
Dervic et al.^66^	Evaluation of a research skills training program for Austrian child and adolescent psychiatry residents (sample size not reported)	The program provided 11 seminars during the academic year, including research/educational career planning, research methods, skills acquirement, ethics committee applications, and grant proposals. As well they offered mentorship and project opportunities	The first responses from the residents were positive, indicating overall satisfaction with the program. However, more detailed feedback or outcomes were not available
Back et al.^67^	Evaluation of the DART program Medical University of South Carolina psychiatry residents (sample size not reported)	DART, an NIH-funded research track, provides funding, training, and mentorship to train residents in all phases of research—didactic seminars covering skill development, dissemination, responsible conduct, conferences, and grantsmanship	Most DART graduates reported high satisfaction with the program components and pursued academic careers to continue research activities
Balon et al.^68^	Evaluation of a research colloquium for junior psychiatry researchers from the United States and abroad (*n* = 377)	The research colloquium, funded by the APA Committee on Research Training, offers a 2-day research conference, pairing trainees with mentors and providing a forum for research presentations. They also provide the trainees with longitudinal follow-up and a stipend to help offset travel costs	Broadly, colloquium participants reported a positive overall satisfaction with the program, high transition to research-based careers, extramural funding from various sources, and high numbers of papers and presentations
Duthie et al.^69^	Evaluation of an annual research symposium for Scottish psychiatric trainees (*n* = 22)	Authors surveyed symposium attendants, compiled a list of all presentations to the symposium from 2007 through 2009, and searched major research databases for participants’ publication activity	Nearly 50% of research projects presented at the symposium achieved eventual publication. In addition, feedback from symposia attendees was almost universally positive
Hipolito et al.^70^	Evaluation of a summer research training program for Dartmouth and Howard Universities’ psychiatry residents (*n* = 77)	The program received a 5-year grant to explore mental health treatment and research cultural factors. The authors conducted questionnaires to evaluate participant experiences	Participants reported overall satisfaction and enhanced confidence in dealing with minority patients and the diverse factors affecting their research treatment
Mezzacappa et al.^71^	Evaluation of a research scholarship program for U.S. child and adolescent fellow (*n* = 52)	The program included protected time for scholarly pursuits and a mentorship program to support academic interests	After implementing the program changes, there was a tenfold increase in the number of residents engaged in scholarly pursuits
Tsai et al.^72^	Evaluation of a research training program for University of California psychiatry residents (*n* = 48)	Program components include mentorship, individualized training plans, clinical and research experiences, protected research time, and funding	More than 80% matriculated to postdoctoral research fellowships, irrespective of previous training
Zisook et al.^73^	Evaluation of AADPRT-led research training conferences (*n* = 284)	AADPRT directors led several full-day seminars to increase residents’ competence in evidence-based medicine, research literacy, and knowledge translation	Feedback from the program’s first 5 years documented excellent attendance, perceived satisfaction, and material usefulness
Bhat et al.^74^	Description and informal evaluation of three Canadian RT programs (*n* = 44)	Three RT programs at the University of British Columbia, the University of Toronto, and McGill University provided participants with protected time, seminars, opportunities to pursue graduate degrees, and funding for research	Although formal outcomes were not reported, this study provides the only published research on Canadian RT programs. In addition, the authors outline some of the issues with psychiatry resident research documentation in the literature
Goldstein et al.^75^	Evaluation of a NIMH-funded research training program for U.S. psychiatry residents from minority populations (*n* = 99)	The PMRTP aimed to support psychiatric investigators from underrepresented populations in developing and maintaining research careers	Nearly 70% reported publishing their research findings, and 64% received post-training research grants; most pursued academic careers
Posporelis et al.^76^	Evaluation of two research training programs for Johns Hopkins psychiatry residents (*n* = 11)	The two training curricula separately targeted first-year and 2nd-to-4th-year psychiatry residents, involving protected research time, mentorship, and seminars	Overall, participants reported satisfaction with both programs and increased interest in academic research careers after exposure to the curricula
Himelhoch et al.^77^	Evaluation of a systematic review and meta-analysis course for University of Maryland psychiatry residents (*n* = 54)	The authors developed, implemented, and evaluated an interactive, web-enhanced course to provide tools for conducting and reporting systematic reviews and meta-analyses	There was overall participant satisfaction and improved confidence with reviews. Academic productivity included 11 presentations, four publications, and two awards
Aftab et al.^78^	Evaluation of a resident-led research newsletter for Cleveland psychiatry residents (*n* = 29)	The authors developed, implemented, and evaluated a resident-led psychiatry research newsletter (“Research Watch”) to showcase resident research activity	Nearly 50% reported improved interest in scholarly activities, current psychiatric research, and participation in research projects
Besterman et al.^79^	Evaluation of a biennial research retreat for California psychiatry residents (*n* = 65)	The authors developed, implemented, and evaluated a biennial psychiatry resident research retreat to enhance research networking and mentorship	Overall participant satisfaction and improvement over consecutive years of the retreat, particularly for networking sessions
Campbell et al.^80^	Evaluation of the DART program for Medical University of South Carolina psychiatry residents (*n* = 132)	DART, an NIH-funded research track, provides funding, training, and mentorship. Didactic seminars covered research skills, manuscript writing, dissemination, ethics, and grantsmanship	Most DART graduates reported high satisfaction with the program components and pursued academic careers to continue research activities
Viswanath et al.^81^	Evaluation of an ethics training module for the Indian NIMH psychiatry residents (*n* = 25)	The authors developed, implemented, and evaluated a new research ethics training module for psychiatry residents (“The Five-Tier Approach”)	Participants demonstrated an adequate understanding of ethical concepts, including autonomy, benefits, and justice
Calhoun et al.^82^	Controlled evaluation of an integrated research training program for Yale child psychiatry trainees (*n* = 36)	The AJSP aimed to prepare child psychiatry clinician scientists by providing dedicated funding, protected research time, and mentorship to trainee participants	Compared to controls, AJSP participants were more likely to hold AACAP certification and have more awards, publications, and grants

AACAP: American academy of child and adolescent psychiatry; AADPRT: American association of directors of psychiatric residency training; AJSP: Albert J. Solnit integrated training program; APA: American psychiatric association; DART: drug abuse research training; NIMH: national institute of mental health; PART: psychiatrists acquiring research training; PMRTP: program for minority research training program in psychiatry; RRTP: resident research training program; RT: research track; U.S.: United States.

### Study quality

Using the JBI instrument, 34 of the 36 studies received low-quality ratings (Table 3). Most intervention-type studies reported numerous positive outcomes (e.g., grants, publications, matriculation to research roles) based on retrospective analyses but without any formal comparison to baseline productivity before implementing the program or to an external comparison group; only two of the studies^64,82^ included a control group.

**Table 3.** Joanna Briggs Institute’s risk of bias assessments.

Study	Were the criteria for inclusion in the sample clearly defined?	Were the study subjects and the setting described in detail?	Was the exposure measured validly and reliably?	Were objective, standard criteria used for the measurement of the condition?	Were confounding factors identified?	Were strategies to deal with confounding factors stated?	Were the outcomes measured validly and reliably?	Overall appraisal
Hershberg et al.^47^	Yes	Yes	No	N/A	Yes	No	No	Low
Strauss et al.^48^	Yes	Yes	No	N/A	Yes	No	No	Low
Andrews et al.^49^	Yes	Yes	No	N/A	Yes	No	No	Low
Balon and Singh^50^	Yes	Yes	No	N/A	Yes	No	No	Low
Fitz-Gerald et al.^51^	Yes	Yes	No	N/A	Yes	Yes	No	Low
Winter et al.^52^	Yes	Yes	No	N/A	Yes	No	No	Low
Silberman et al.^53^	Yes	Yes	No	N/A	Yes	No	No	Low
Shanmugalingam et al.^54^	Yes	Yes	No	N/A	Yes	No	No	Low
Laliberté et al.^55^	Yes	Yes	No	N/A	Yes	Yes	No	Low
MacMaster et al.^56^	Yes	Yes	No	N/A	Yes	No	No	Low
Fisher and Bender^57^	Yes	Yes	No	N/A	Yes	No	No	Low
Tuma et al.^58^	Yes	Yes	No	N/A	Yes	No	No	Low
Belmont et al.^59^	Yes	Yes	No	N/A	Yes	No	No	Low
Balon and Kuhn^60^	Yes	Yes	No	N/A	Yes	No	No	Low
Halpain et al.^61^	Yes	Yes	No	N/A	Yes	No	No	Low
Gilbert et al.^62^	Yes	Yes	No	N/A	Yes	No	No	Low
Kunik et al.^63^	Yes	Yes	No	N/A	Yes	No	No	Low
Löwe et al.^64^	Yes	Yes	Yes	N/A	Yes	Yes	Yes	High
Roane et al.^65^	Yes	Yes	No	N/A	Yes	No	No	Low
Dervic et al.^66^	Yes	Yes	No	N/A	Yes	No	No	Low
Back et al.^67^	Yes	Yes	No	N/A	Yes	No	No	Low
Balon et al.^68^	Yes	Yes	No	N/A	Yes	No	No	Low
Duthie et al.^69^	Yes	Yes	No	N/A	Yes	No	No	Low
Hipolito et al.^70^	Yes	Yes	No	N/A	Yes	No	No	Low
Mezzacappa et al.^71^	Yes	Yes	No	N/A	Yes	No	No	Low
Tsai et al.^72^	Yes	Yes	No	N/A	Yes	No	No	Low
Zisook et al.^73^	Yes	Yes	No	N/A	Yes	No	No	Low
Bhat et al.^74^	Yes	Yes	No	N/A	Yes	No	No	Low
Goldstein et al.^75^	Yes	Yes	No	N/A	Yes	No	No	Low
Posporelis et al.^76^	Yes	Yes	No	N/A	Yes	No	No	Low
Himelhoch et al.^77^	Yes	Yes	No	N/A	Yes	No	No	Low
Aftab et al.^78^	Yes	Yes	No	N/A	Yes	No	No	Low
Besterman et al.^79^	Yes	Yes	No	N/A	Yes	No	No	Low
Campbell et al.^80^	Yes	Yes	No	N/A	Yes	No	No	Low
Viswanath et al.^81^	Yes	Yes	No	N/A	Yes	No	No	Low
Calhoun et al.^82^	Yes	Yes	Yes	N/A	Yes	Yes	Yes	High

### Synthesis of findings from the survey studies

While participation in scholarly research, quality improvement, or educational projects relevant to psychiatry is required in most residency programs, requirements vary significantly. Few require any direct research experience for residency completion. For example, a 2014 survey of representatives of all 17 accredited Canadian residency programs found that among the 11 programs mandating research requirements, qualifying activities ranged from fully independent resident-led research projects to quality improvement initiatives, assisting with faculty research, or simply presenting research.^54^ However, none required publication of the resident’s final work.^54^

Although most programs offer some form of research activity (e.g., at least presenting an article at a journal club), earlier survey studies demonstrated minimal research resources were available to residents.^47,48,50,51^ The research infrastructure for psychiatry residents—exposure to psychiatry research, availability of resources, training, protected time, and dedicated funding—varied substantially across programs.^54–56^ Epidemiological methods, critical appraisal skills, ethical issues, and research design were the most commonly taught topics in didactic resident seminars.^49^ Residents and training directors consistently identified the need for protected research time and support as the most valued resources.^54–56^ Agreeing on a research training plan with a mentor and regularly reviewing support and supervision through a newly developed mentoring process was associated with high trainee satisfaction levels.^52^ While early exposure to research was initially thought to promote future research interest,^55^ two national surveys of Canadian psychiatry residents have challenged this assumption, suggesting that most residents lose interest in research by the time they graduate and that participation in a research track does not appear to enhance research interest.^55,56^

In summary, survey studies indicated that while most programs offered some research activity, the availability of resources (e.g., protected time), the content of didactic seminars, and the extent of research requirements varied substantially across programs. There were also challenges with enhancing research interest among residents, particularly those who lack pre-residency research training experience.

### Synthesis of findings from the intervention studies

Across studies, several identified various mechanisms by which psychiatry residents could receive additional research training. At the highest end, we identified several externally funded, comprehensive research training programs which offer formal training opportunities (i.e., graduate degrees and fellowships) integrated with psychiatry residency training. Most of these higher-end programs are available to U.S.-based psychiatry residents and are funded through the National Institutes of Health (NIH) or the National Institutes of Mental Health (NIMH). These programs generally demonstrated good outcomes and appeared to improve the scientific workforce, promote future research involvement, and generate awards, publications, and grants.^57,67,75,80,82^ For example, Yale’s Albert J. Solnit Integrated Training Program (AJSP), a 15-year NIMH-funded initiative, improved future professional board affiliation and generated more awards, publications, and grant funding than a control group.^82^ In addition, Canadian psychiatry residents can apply for a similar offering through the RCPSC’s Clinician Investigator Program (CIP), which provides at least 2 years of protected research time and salary support, enabling residents to pursue a thesis-based graduate degree alongside their clinical training.^83^ The CIP has been very successful, and most graduates who have gone through the program have become quite involved in research—although not all.^84,85^

In contrast to these formal programs, several locally developed research tracks offered more flexible, individualized, and innovative options to facilitate resident entry into research careers.^59,60,62–66,71,72,74,76^ For instance, shorter research training opportunities, such as intensive summer research institutes or symposia, targeted residents with less formal research interests, with attendants reporting high satisfaction.^58,61,68–70,73,79^ Similarly, 3-day NIMH-funded psychiatry research conferences, which included morning plenary sessions and afternoon small-group teaching sessions, appeared to consolidate knowledge and provide practical research skills for resident participants.^86^ For child and adolescent psychiatry trainees, designated research workshops encouraged child psychiatrists who wished to pursue a research career and effectively encouraged trainee research.^87^

There were also three miscellaneous research training interventions. The first was a novel online research ethics training module for psychiatry residents, which incorporated five modules for teaching psychiatry residents about the basics of ethical concepts, including autonomy, benefits, and justice.^81^ The second was a systematic review and meta-analysis course, developed using an interactive, web-enhanced module that gave trainees an overview of the essential tools for conducting and reporting systematic reviews and meta-analyses, overall participant satisfaction, and improved confidence with reviews.^77^ Finally, a resident-led research newsletter showcasing resident research activity was associated with a nearly 50% improvement in research interest and participation in research projects among resident participants.^78^

Given the lack of evidence in the literature, we felt that including some expert opinion could be worthwhile, and consequently, offering some practical advice could be helpful. Based on these findings and our own experience, we compiled a list of resources, strategies, and “tips” for psychiatry residents interested in research (Appendix 4).

## Discussion

### Summary of findings

The present study is the first comprehensive review of research training for psychiatry residents. Our review found 36 studies describing the state of psychiatry resident research opportunities and evaluating diverse research training methods. Formal research training programs enhanced the scientific workforce consistently, promoted future research involvement, and generated awards, publications, and grants. However, as these programs might simply sustain the scholarship of residents with previous research experience and interest, they may not appeal to trainees with less research interest or those who foresee non-research-based careers.

While there is insufficient evidence to inform guidelines for organizing research training programs concerning national requirements, we offer suggestions based on the available information.^74^ Different intervention approaches studied may help inform how Canadian residency training programs can develop a more uniform approach to meeting the CanMEDS Scholar role for research. To help set up a reliable standard, a pragmatic recommendation would be for all training programs to teach critical appraisal and presentation skills. As this EPA occurs early in training within the FoD stage, relevant teaching to achieve this EPA should co-occur during the corresponding phase of training (i.e., PGY1&2). For example, in-person research training workshops or journal clubs^88^ or online training (i.e., the United Kingdom’s Critical Appraisal Skills Program) could address critical appraisal content using checklists to help evaluate critical appraisal skill competence when residents present.^89^

In parallel, all Canadian psychiatry residency training programs should have a research requirement, yet only 11 of the 17 accredited programs reported including research requirements.^54^ At a minimum, Canadian psychiatry residents should complete a review of a major psychiatric topic applicable to their future practice and present it formally at grand rounds. The proposed requirement would demonstrate expertise in an area residents plan to practice and distinguish them from their peers for practice placement.

In a parallel vein, it is interesting to contrast the training of psychiatry residents with related mental health professionals’ training. For example, a previous study compared the training of psychologists and psychiatrists, concluding that the latter have little formal training in studying human behavior and little contact with problems and methods in science. In contrast, the former has a more extensive background in studying human behavior and formal training in science.^90^ To that end, another study suggested that there may be mutual benefit from academic collaboration across institutions to achieve their respective educational and training missions.^91^

#### Strengths and limitations

While augmenting the current research-oriented EPAs may be beyond the scope of this article, we believe this article has several strengths. First, based on existing literature, it describes the components and sophistication of critical appraisal and presentation skills that psychiatry residents need to meet for entrusting the related EPA. These descriptions provide Canadian programs with tangible suggestions for base program research requirements. Second, our review’s findings identify the importance of distinguishing research capability and development levels for different residents. Different tracks could be available (i.e., basic for the general clinician, enhanced for the academic clinician, and advanced for the clinician-scientist/research track academic).

However, a few limitations call for further discussion. First, as our search strategy focused on publications that focus on psychiatry, we excluded reviews on more general approaches. To address this, we included additional data on the RCPSC’s CIP from a 2011 review published by Hayward et al. (performed by the RCPSC 13 years after the institution of the CIP in 1995) and a 2010/2011 survey on Canadian CIP programs by Hayward et al.,^85^ as these indirectly provided some coverage of the psychiatry programs that were involved. Finally, as the CIP has been a considerable success and some coauthors have had personal experience with this excellent RCPSC program, we discussed it in our review.

Second, despite the breadth of findings, the included studies’ quality was poor due to few controls and standardized measurements. Given the uncontrolled designs of most studies, the presented findings are highly susceptible to selection bias. Most research tracks and formal training programs cater to motivated trainees.^53^ Even the two high-quality studies^64,82^ had severe methodological limitations in selecting controls (i.e., these groups were not randomly allocated).

Third, no intervention studies or surveys addressed critical appraisal and presentation skills to inform Canadian psychiatry residency training programs about a standard to meet this EPA in the current CBD framework. There were few Canadian-based intervention studies, and most recommendations were extrapolated to Canadians.

A fourth limitation was the high heterogeneity in reported outcomes (precluding quantitative synthesis) and methods across studies—some studies not fully delineating the feasibility or effectiveness of their interventions and few measuring sustained competence over a long-term follow-up period were another limitation of our review.

## Conclusions

In conclusion, our systematic review has shed light on the challenges of integrating research training within the CBD framework in psychiatry. The interventions described in the included studies primarily focused on cultivating productive, independent researchers, while CBD emphasizes the development of research basics, such as critical appraisal and research fundamentals, alongside clinical excellence.

Drawing insights from other successful CBD models, we propose that psychiatry research training could incorporate one or two general EPAs applicable to all residents, emphasizing foundational research competencies. Additionally, specialized or enhanced EPAs could be designed for residents pursuing research tracks or fellowships, allowing them to deepen their research expertise.

Establishing core metrics for evaluating research competency across Canadian psychiatry residency training programs is crucial. By doing so, the RCPSC and the Canadian Psychiatric Association can promote a standardized and comprehensive approach to research training, aligned with the principles of CBD.

In light of the limited evidence available in the literature, we hope that our compilation of resources, strategies, and tips for psychiatry residents interested in research will serve as a valuable, practical element of this article. These resources aim to empower aspiring researchers and contribute to the overall enhancement of research training within psychiatry residency programs.

While integrating research training within CBD presents challenges, we believe that our recommendations and the proposed framework will foster a culture of research excellence among psychiatry residents and contribute to advancing the field of psychiatric research in Canada.

## Supplemental Material

sj-docx-1-smo-10.1177_20503121231216846 – Supplemental material for Assessing research competency development in Canadian psychiatry residency programs: A systematic review and future directions Click here for additional data file. Supplemental material, sj-docx-1-smo-10.1177_20503121231216846 for Assessing research competency development in Canadian psychiatry residency programs: A systematic review and future directions by Anees Bahji, Marlon Danilewitz, David Crockford, Nicholas Delva and Benjamin Rusak in SAGE Open Medicine
