# Supplementary material for: Assessing research competency development in Canadian psychiatry residency programs: A systematic review and future directions
Source: SAGE Open Med. 2023 Dec 12;11:20503121231216846. doi: 10.1177/20503121231216846 (PMC10722931; doi:10.1177/20503121231216846)
Supplement: sj-docx-1-smo-10.1177_20503121231216846 – Supplemental material for Assessing research competency development in Canadian psychiatry residency programs: A systematic review and future directions [file sj-docx-1-smo-10.1177_20503121231216846.docx]

**Appendix 1.** Royal College of Physicians and Surgeons of Canada Psychiatry Research Competencies, adapted from 2020 guidelines [36].

## OVERVIEW

- Upon completing training, a resident is expected to be a competent psychiatrist specialist, assuming a consultant’s role in the specialty. In addition, the resident must acquire a working knowledge of the theoretical basis of Psychiatry, including its foundations in the basic medical sciences and research.

## SCHOLAR

- As Scholars, psychiatrists demonstrate a lifelong commitment to excellence in practice through continuous learning, teaching others, evaluating evidence, and contributing to scholarship.
- Demonstrate an understanding of the scientific principles of research and scholarly inquiry and the role of research evidence to:
  - Integrate the best available evidence into practice
  - Recognize practice uncertainty and knowledge gaps in clinical and other professional encounters and generate focused questions that can address them
  - Identify, select, and navigate pre-appraised resources
  - Critically evaluate the integrity, reliability, and applicability of health-related research and literature
  - Integrate evidence into decision-making in their practice
- Contribute to the creation and dissemination of knowledge and procedures applicable to health
- Describe the principles of research and scholarly inquiry
- Describe the principles of research ethics
- Pose a scholarly question
- Conduct a systematic search for evidence
- Select and apply appropriate methods to address the question
- Disseminate the findings of a study
- Participate in scholarly research, quality insurance, or educational project relevant to psychiatry, demonstrating primary responsibility for at least one of the following elements of the project:
  - Development of the hypothesis, which must include a comprehensive literature review
  - Development of the protocol for the scholarly project
  - Preparation of a grant application
  - Development of the research ethics proposal
  - Interpretation and synthesis of the results
- Identify ethical principles for research and incorporate them into obtaining informed consent, considering potential harms and benefits, and considering vulnerable and marginalized populations
  - Adhere to guidelines for ethical research, including obtaining valid consent, where appropriate, lack of coercion, and avoidance of harm.
  - Contribute to the work of a research program.
  - Pose questions amenable to scholarly investigation and select appropriate methods to address them.
  - Conduct scholarly work, including research, quality assurance, or educational initiatives.
  - Summarize and communicate relevant research and scholarly inquiry findings to professional and lay audiences, including patients and their families.

## PROFESSIONAL

- Abide by accepted guidelines concerning research, education, and clinical care, including those that pertain to ethical interactions with industry, especially the pharmaceutical industry

## COLLABORATOR

- Work with and learn from others to assess, plan, and review other tasks, such as research problems.

**Appendix 2.** PRISMA Checklist.

| **Section and Topic** | **Item #** | **Checklist item** | **Location where item is reported** |
| --- | --- | --- | --- |
| **TITLE** | | |  |
| Title | 1 | Identify the report as a systematic review. | 1 |
| **ABSTRACT** | | |  |
| Abstract | 2 | See the PRISMA 2020 for Abstracts checklist. | 2-3 |
| **INTRODUCTION** | | |  |
| Rationale | 3 | Describe the rationale for the review in the context of existing knowledge. | 4 |
| Objectives | 4 | Provide an explicit statement of the objective(s) or question(s) the review addresses. | 5 |
| **METHODS** | | |  |
| Eligibility criteria | 5 | Specify the inclusion and exclusion criteria for the review and how studies were grouped for the syntheses. | 6 |
| Information sources | 6 | Specify all databases, registers, websites, organizations, reference lists and other sources searched or consulted to identify studies. Specify the date when each source was last searched or consulted. | 7 |
| Search strategy | 7 | Present the full search strategies for all databases, registers and websites, including any filters and limits used. | 7 |
| Selection process | 8 | Specify the methods used to decide whether a study met the review's inclusion criteria, including how many reviewers screened each record and each report retrieved, whether they worked independently, and, if applicable, details of automation tools used in the process. | 8 |
| Data collection process | 9 | Specify the methods used to collect data from reports, including how many reviewers collected data from each report, whether they worked independently, any processes for obtaining or confirming data from study investigators, and, if applicable, details of automation tools used in the process. | 8 |
| Data items | 10a | List and define all outcomes for which data were sought. Specify whether all results that were compatible with each outcome domain in each study were sought (e.g. for all measures, time points, analyses), and if not, the methods used to decide which results to collect. | 8 |
|  | 10b | List and define all other variables for which data were sought (e.g. participant and intervention characteristics, funding sources). Describe any assumptions made about any missing or unclear information. | 8 |
| Study risk of bias assessment | 11 | Specify the methods used to assess risk of bias in the included studies, including details of the tool(s) used, how many reviewers assessed each study and whether they worked independently, and if applicable, details of automation tools used in the process. | 8 |
| Effect measures | 12 | Specify for each outcome the effect measure(s) (e.g., risk ratio, mean difference) used in synthesizing or presenting results. | 9 |
| Synthesis methods | 13a | Describe the processes used to decide which studies were eligible for each synthesis (e.g. tabulating the study intervention characteristics and comparing against the planned groups for each synthesis (item #5)). | N/A |
|  | 13b | Describe any methods required to prepare the data for presentation or synthesis, such as handling missing summary statistics or data conversions. | N/A |
|  | 13c | Describe any methods used to tabulate or visually display the results of individual studies and syntheses. | N/A |
|  | 13d | Describe any methods used to synthesize results and provide a rationale for the choice(s). If meta-analysis was performed, describe the model(s), method(s) to identify the presence and extent of statistical heterogeneity, and software package(s) used. | N/A |
|  | 13e | Describe any methods to explore possible causes of heterogeneity among study results (e.g. subgroup analysis, meta-regression). | N/A |
|  | 13f | Describe any sensitivity analyses conducted to assess the robustness of the synthesized results. | N/A |
| Reporting bias assessment | 14 | Describe any methods used to assess the risk of bias due to missing results in a synthesis (arising from reporting biases). | N/A |
| Certainty assessment | 15 | Describe any methods used to assess certainty (or confidence) in the body of evidence for an outcome. | N/A |
| **RESULTS** | | |  |
| Study selection | 16a | Describe the search and selection process results, from the number of records identified in the search to the number of studies included in the review, ideally using a flow diagram. | 10 |
|  | 16b | Cite studies that might appear to meet the inclusion criteria but which were excluded, and explain why they were excluded. | N/A |
| Study characteristics | 17 | Cite each included study and present its characteristics. | 10 |
| Risk of Bias in studies | 18 | Present assessments of risk of bias for each included study. | 10 |
| Results of individual studies | 19 | For all outcomes, present, for each study: (a) summary statistics for each group (where appropriate) and (b) an effect estimate and its precision (e.g. confidence/credible interval), ideally using structured tables or plots. | 10-13 |
| Results of syntheses | 20a | For each synthesis, briefly summarise the characteristics and risk of bias among contributing studies. | N/A |
|  | 20b | Present results of all statistical syntheses conducted. If meta-analysis was done, present for each the summary estimate and its precision (e.g. confidence/credible interval) and measures of statistical heterogeneity. If comparing groups, describe the direction of the effect. | N/A |
|  | 20c | Present results of all investigations of possible causes of heterogeneity among study results. | N/A |
|  | 20d | Present results of all sensitivity analyses conducted to assess the robustness of the synthesized results. | N/A |
| Reporting biases | 21 | Present assessments of risk of bias due to missing results (arising from reporting biases) for each synthesis assessed. | N/A |
| Certainty of evidence | 22 | Present assessments of certainty (or confidence) in the body of evidence for each outcome assessed. | N/A |
| **DISCUSSION** | | |  |
| Discussion | 23a | Provide a general interpretation of the results in the context of other evidence. | 14 |
|  | 23b | Discuss any limitations of the evidence included in the review. | 15-16 |
|  | 23c | Discuss any limitations of the review processes used. | 15-16 |
|  | 23d | Discuss implications of the results for practice, policy, and future research. | 14-17 |
| **OTHER INFORMATION** | | |  |
| Registration and protocol | 24a | Provide registration information for the review, including register name and registration number, or state that the review was not registered. | 6 |
|  | 24b | Indicate where the review protocol can be accessed, or state that a protocol was not prepared. | 6 |
|  | 24c | Describe and explain any amendments to information provided at registration or in the protocol. | N/A |
| Support | 25 | Describe sources of financial or non-financial support for the review, and the role of the funders or sponsors in the review. | 1-2 |
| Competing interests | 26 | Declare any competing interests of review authors. | 1-2 |
| Availability of data, code and other materials | 27 | Report which of the following are publicly available and where they can be found: template data collection forms; data extracted from included studies; data used for all analyses; analytic code; any other materials used in the review. | N/A |

**Appendix 3.** Search syntaxes

**EMBASE:** inception to March 17, 2021

| Order | Search | Hits |
| --- | --- | --- |
| 1 | research training.mp | 2727 |
| 2 | research curriculum.mp | 158 |
| 3 | resident research.mp | 309 |
| 4 | exp medical education/ or resident education.mp. or exp residency education/ or exp curriculum/ | 379646 |
| 5 | exp psychiatry/ or psychiatry resident.mp | 133051 |
| 6 | 1 or 2 or 3 | 3095 |
| 7 | 4 and 5 and 6 | 55 |
| 8 | limit 7 to (human and English language) | 35 |

**MEDLINE:** inception to March 17, 2021

| Order | Search | Hits |
| --- | --- | --- |
| 1 | research training.mp | 2268 |
| 2 | research curriculum.mp | 122 |
| 3 | resident research.mp | 247 |
| 4 | exp Curriculum/ or exp "Internship and Residency"/ or exp Clinical Competence/ or resident education.mp. or exp Education, Medical, Graduate/ | 216,861 |
| 5 | exp Psychiatry/ or psychiatry resident.mp | 105366 |
| 6 | 1 or 2 or 3 | 2550 |
| 7 | 4 and 5 and 6 | 37 |
| 8 | limit 7 to (human and English language) | 27 |

**PsycINFO:** inception to March 17, 2021

| Order | Search | Hits |
| --- | --- | --- |
| 1 | research training.mp | 1466 |
| 2 | research curriculum.mp | 82 |
| 3 | resident research.mp | 13 |
| 4 | exp Medical Residency/ or exp Medical Education/ or exp Curriculum Development/ or exp Curriculum/ | 145977 |
| 5 | exp Psychiatric Training/ or exp Psychiatrists/ or exp Psychiatry/ or psychiatry resident.mp | 63520 |
| 6 | 1 or 2 or 3 | 1552 |
| 7 | 4 and 5 and 6 | 74 |
| 8 | limit 7 to (human and English language) | 71 |

**ERIC (EBSCO):** inception to March 17, 2021

| Order | Search | Hits |
| --- | --- | --- |
| 1 | Psychiatry Education | 2100 |
| 2 | Research training | 78,220 |
| 3 | 1 and 2 | 145 |
| 4 | Limit 3 to (English) | 131 |
| 5 | exp Psychiatric Training/ or exp Psychiatrists/ or exp Psychiatry/ or psychiatry resident.mp | 63520 |
| 6 | 1 or 2 or 3 | 1552 |
| 7 | 4 and 5 and 6 | 74 |
| 8 | limit 7 to (human and English language) | 71 |

**Appendix 4.** Compilation of research tips, strategies, and resources for psychiatry residents.

## Value of Psychiatric Research

The gap between the need for and availability of psychiatrists involved in research continues to grow. Some identify the urgency of incorporating clinical and translational sciences into research training for psychiatry residents [5]. Despite the increase in the number of MD/PhDs entering psychiatry, these numbers remain low, and there remains an urgent need for more clinician-scientists in psychiatry [92,93]. While it remains unclear how to encourage more psychiatric trainees to pursue meaningful research careers, research training during residency offers many potential benefits [94]. Learners can gain in-depth knowledge about some aspects of psychiatry, understand the basic notions of research, and gain analytical and technical skills [95]. Importantly, research training often gives opportunities to present one's work at conferences, which encourages residents to value their efforts and provides the satisfaction of contributing to the advancement of the field [95].

## Frameworks

In the CBME era, competency-based frameworks can help residents gain particular research skill sets [4,7,96]. They have also proposed specific research training models for psychiatry subspecialties—such as child and adolescent psychiatry [25,66]—and under-represented groups in psychiatry [48,75,97,98]. In addition, one- or two-day research colloquia for junior investigators, such as those organized by the Canadian and American Psychiatric Associations, create opportunities for residents to share their work and engage in peer mentorship and often help defray travel and registration costs for trainees [68,99].

## Start Early

The mounting interest in psychiatric specialties has forced a critical re-evaluation of training opportunities for interested candidates and the reappraisal of mechanisms to balance scholarly activities with clinical demands [95]. Starting research efforts early can help residents plan, enabling a balance between clinical duties and research activities [95]. Engaging psychiatry residents in research as early as possible in their training is crucial for subsequent research activity. Residents should view research as a sub-specialization within psychiatry that will require residents to dedicate time and identify many resources to be successful.

## Think About the Kind of Researcher You Want To Be

As there are many types and styles of research, trainees should consider the kind of research they want to do, which will inform their path to becoming successful researchers. For example, one can support research indirectly by helping colleagues recruit participants for trials or conducting research intake surveys with patients. One can also be a good research consumer and critically appraise research publications. At the other extreme, one can become a full-time clinician-scientist researcher. In parallel, trainees must ask program or research directors about the research requirements needed for graduation, available funding, and protected research time.

## Ask About Protected Time

Having an uninterrupted period to focus on research is crucial to research success. Some residents may have more than 20% protected time if they commit to "research tracks," while other arrangements, such as four-week "research blocks," are often available for most residents [62]. Besides procuring protected research time to keep a competitive edge as a psychiatry researcher, resources should tailor to different research involvement levels. Those envisioning a primarily research-oriented career will need more protected research time and resources; those satisfied with being research consumers require less specific research investments. For select residents who demonstrate an early interest in research rather than all trainees, there may be value towards enrolment or offerings of “research tracks.”

## Find A Mentor

Understanding the basics of identifying a mentor is another significant first step toward establishing yourself as a psychiatry researcher [100]. When considering potential mentors, residents may consider both content (e.g., someone well-versed in a specific clinical domain, such as mood disorders) and methodological mentors (e.g., research tools or analytic techniques). Seeking mentors may lead to future partnerships and collaborations with research groups. Funding agencies, professional societies, and training institutions increasingly favour groups of researchers over solo endeavours, which encourages collaboration. While resident researchers may not initially be group leaders, perseverance and hard work will generally lead to increased responsibilities and leadership with time. Partnerships among funding agencies, professional societies, and training institutions can help lay the groundwork for the next generation of psychiatrists to stay on the path to rewarding scientific research careers [100].

## Familiarize Yourself with Your Residency Program’s Research Supports

Most psychiatry departments have designated research directors and deputy's heads of research tasked with trainees' mentoring and guidance throughout their residencies. Psychiatry research administrative staff can also support psychiatry resident research efforts. For example, the staff is often very familiar with local financial support within the university. They can point residents toward specific funding for their projects or stage of training. Administrative support is essential, which can help coordinate research ethics board applications, grant applications, manage accounts, access physical space, secure other resources, and identify secure data storage. Effective communication with organizational leadership can help residents appreciate the long-term stability of research projects in their institutions—specifically, what they can expect financially and administratively in the coming years.

## Psychological Requirements

While a research career in psychiatry can be highly satisfying, it is not easy. Psychiatry residents who have an interest in pursuing a research-based career face many obstacles, including little exposure to psychiatry research in medical school, long clinical duty hours, limited protected research time, scarcity of peer research mentors, lack of funding opportunities, and inadequate training in the statistical aspects of psychiatric research [94,95,101]. Research can be frustrating, requiring high tolerance for frequent rejection, effective time management skills, and work-life balance with clinical responsibilities. Nevertheless, supportive mentors, resilience, and a strong inner drive can motivate residents to become successful researchers despite formidable obstacles [102,103].

## Training

There are also various courses and guided readings for those pursuing formal research training to acquire research skills. Psychiatry departments have increasingly integrated formal research training pathways, hierarchies, and tailored infrastructure to develop research competence. For psychiatry trainees, formal training programs, including the MD/Ph.D. stream for medical students or the RCPSC Clinician Investigator Program and research fellowships for residents, can provide more in-depth training. These programs aim to have psychiatry residents graduate with minimal research competence, while others may go above and beyond. Select psychiatry residents will pursue formal training in a research discipline. A significant advantage to participating in these traditional training programs is access to mentorship and expertise in research methods and analytics for learners at undergraduate, postgraduate, fellowship, and internship levels. Psychiatry-oriented graduate programs offer various coursework offerings in participatory research, neuroimaging, psychotherapy research, early intervention in psychiatry, personalized psychiatry, skill development, critical appraisal, scientific writing, and biostatistics. Fortunately, there are many excellent training opportunities for psychiatry residents. In Canada, there are research-based MSc and Ph.D. in Psychiatry programs available through Dalhousie University [104], McGill University [105], and the University of Alberta [106]. It remains to be seen how these programs will affect resident involvement in research and whether their design may need reconsidering to attract more residents. However, there are many more formal training pathways for psychiatric research in the United States.

For example, Columbia University offers several subspecialties, and postgraduate research fellowships, through the NIMH [107]. In addition, motivated trainees may seek to foster international collaboration with colleagues in Europe or other parts of the world. While every department may not have the resources or the personnel to mount internal graduate programs, they can provide financial support for residents to acquire graduate degrees through related departments or point them to external agencies, such as the Royal College. But how effective are formal research tracks for promoting clinician-scientist careers post-residency? In one study, 80% of graduates who completed a research track in their residency program later matriculated to a postdoctoral research fellowship—irrespective of their previous doctoral-level training in the basic or social sciences. Promising results from another longitudinal study of research track programs suggest that it is possible to encourage academic research careers using peer mentoring. This innovative approach requires minimal funding, causes little disruption to the residents' schedule, and engages the gamut of individuals involved in psychiatry care and research [76].

## Funding Opportunities

As funding can be a rate-limiting step to research, awareness of the relevant, available funding organizations is critical. Many funding strategies exist for pursuing research for Canadian and American psychiatric trainees.

1. The Royal College of Physicians and Surgeons of Canada offers the Karen Mann Catalyst Grant for Medical Education Research [108].
2. The Physician Services Institute Foundation funds Ontario residents in all disciplines interested in researching health services, education, or basic sciences [109].
3. The MSI Foundation offers a parallel funding mechanism for Albertan residents [110].
4. The Michael Smith Foundation for Health Research provides academic support for British Columbian trainees and residents [111].
5. The Canadian Institutes of Health Research provides more funding to support psychiatric trainees in various mental health and addiction disciplines [112,113].
6. The Canadian Psychiatric Association also provides funds, grants, and resources for research-oriented residents [114].
7. The American National Institute of Mental Health has several dedicated research education programs and grants for psychiatry residents [115,116].
8. The Brain & Behavior Research Foundation, formerly NARSAD, offers many research grants and funding types, with several “new investigator” awards suited for early-career trainees [117].
9. The American College of Psychiatrists also has designated funding for psychiatry residents [118].
10. The American Psychiatric Association also offers several research-based resources, including several fellowships which have integrated research components [119], a dedicated psychiatry research fellowship [120], additional resources for fellows [121], a research colloquium [122], a database with links to other external funding and grant opportunities [123], as well as an updated list of different awards, competitions and leadership opportunities [124].

## Concluding Remarks

Although research can seem daunting to busy psychiatry residents, research training during residency also offers many potential benefits. Learners can gain in-depth knowledge about some aspects of psychiatry, understand the basic research principles, and gain analytical and technical skills. At the same time, different research disciplines demand various skills and resources to foster success. Common factors involve mentorship, resilience, a supportive milieu, and access to intellectual and practical research resources. However, if one can accept its challenges and frustrations, there are many rewards and satisfaction in a research-oriented career. Training the next generation of clinician-scientists in psychiatry will require a multi-pronged approach to address disparities in research training across sites while remaining mindful of individual resident preferences. As the demand for psychiatric research increases, so does the expectation that graduating psychiatrists be competent in research basics. While research disciplines require other traits to foster success, common factors involve mentorship, resilience, and access to intellectual and practical research resources. The impending changes in the era of CBME create both an opportunity and a challenge to embed these foundations into psychiatry residency programs.

92. Honer WG and Linseman MA. The physician–scientist in Canadian psychiatry. J Psychiatry Neurosci 2004; 29: 49–56.

93. Arbuckle MR, Luo SX, Pincus HA, et al. Trends in MD/PhD graduates entering psychiatry: assessing the physician-scientist pipeline. Acad Psychiatry 2018; 42: 346–353.

94. Torous J and Padmanabhan J. Research by residents: obstacles and opportunities. Asian J Psychiatry 2015; 13: 81–82.

95. Parmar A, Sharma P and Pal A. Research by psychiatry residents in Indian scenario: challenges and opportunities. Asian J Psychiatry 2016; 19: 3.

96. Gruppen L, Frank JR, Lockyer J, et al. Toward a research agenda for competency-based medical education. Med Teach 2017; 39: 623–630.

97. Yager J and Strauss G. Current concerns in psychiatric residency education: education, quality, manpower, and funding. Psychiatr Ann 1986; 16: 338–344.

98. Yager J, Waitzkin H, Parker T, et al. Educating, training, and mentoring minority faculty and other trainees in mental health services research. Acad Psychiatry 2007; 31: 146–151.

99. Kupfer DJ, Schatzberg AF, Dunn LO, et al. Career Development Institute with enhanced mentoring: a revisit. Acad Psychiatry 2016; 40: 424–428.

100. Chung J and Pao M. Stepping stones for psychiatry residents who pursue scientific research careers. Int Rev Psychiatry 2013; 25: 284–290.

101. Zisook S, Balon R, Björkstén KS, et al. Psychiatry residency training around the world. Acad Psychiatry 2007; 31: 309–325.

102. Garfinkel PE, Bagby RM, Schuller DR, et al. Predictors of success and satisfaction in the practice of psychiatry: a preliminary follow-up study. Can J Psychiatry 2001; 46: 835–840.

103. Garfinkel PE, Bagby RM, Schuller DR, et al. Predictors of professional and personal satisfaction with a career in psychiatry. Can J Psychiatry 2005; 50: 333–341.

104. Dalhousie University. Master’s in Psychiatry Research, <https://medicine.dal.ca/departments/department-sites/psychiatry/education/masters-in-psychiatry-research.html> (2020, accessed 25 March 2020).

105. McGill University. Master’s in Psychiatry. Dep. Psychiatry, <https://www.mcgill.ca/psychiatry/education/graduate-program> (2020, accessed April 2010).

106. University of Alberta. Master’s in Psychiatry Research, <https://www.ualberta.ca/psychiatry/education-programs/graduate-program/index.html> (2020, accessed 25 March 2020).

107. Columbia University Department of Psychiatry. Master’s in Psychiatry Research, <https://www.columbiapsychiatry.org/research/research-training> (2017, accessed 25 March 2020).

108. The Royal College of Physicians and Surgeons of Canada. The Dr. Karen Mann Catalyst Grant in Medical Education, <http://www.royalcollege.ca/rcsite/awards-grants/research-funding/karen-mann-research-grant-e> (2020, accessed 31 March 2020).

109. Physician Services Institute Foundation. Funding Programs, <http://www.psifoundation.org/funding-programs/> (2020, accessed 31 March 2020).

110. M.S.I. Foundation, <http://www.msifoundation.ca/> (2009, accessed 15 December 2020).

111. Michael Smith Foundation for Health Research. Funding, <https://www.msfhr.org/funding> (2012, accessed 15 December 2020).

112. Canadian Institutes of Health Research. Canadian Research Initiative in Substance Misuse, <https://crism.ca/> (2020, accessed 31 March 2020).

113. Government of Canada, Canadian Institutes of Health Research. Institute of Neurosciences, Mental Health and Addiction, <https://cihr-irsc.gc.ca/e/8602.html> (2020, accessed 31 March 2020).

114. Canadian Psychiatric Association, <https://www.cpa-apc.org/> (2020, accessed 31 March 2020).

115. National Institute of Mental Health. NIMH Research Education Programs Supporting Psychiatry Residents, <https://www.nimh.nih.gov/funding/training/nimh-research-education-programs-supporting-psychiatry-residents.shtml> (2020, accessed 31 March 2020).

116. National Institute of Mental Health. Mental Health Information, <https://www.nimh.nih.gov/health/index.shtml> (2020, accessed 31 March 2020).

117. Brain and Behavior Research Foundation. Grants, <https://www.bbrfoundation.org/grants-prizes/grants> (2020, accessed 31 March 2020).

118. The American College of Psychiatrists. Other psychiatric, <https://www.acpsych.org/awards/other-psychiatric-awards> (2020, accessed 31 March 2020).

119. American Psychiatric Association. Available APA/APAF fellowships, <https://www.psychiatry.org/residents-medical-students/residents/fellowships/available-apa-apaf-fellowships> (2020, accessed 31 March 2020).

120. American Psychiatric Association. Psychiatric research fellowship, <https://www.psychiatry.org/residents-medical-students/residents/fellowships/available-apa-apaf-fellowships/psychiatric-research-fellowship> (2020, accessed 31 March 2020).

121. American Psychiatric Association. Fellows’ opportunities toolkit, <https://www.psychiatry.org/residents-medical-students/residents/fellowships/additional-opportunities-for-fellows> (2020, accessed 31 March 2020).

122. American Psychiatric Association. Research colloquium for junior psychiatrists, <https://www.psychiatry.org/psychiatrists/practice/research/research-colloquium> (2020, accessed 31 March 2020).

123. American Psychiatric Association. External fellowships and awards, <https://www.psychiatry.org/residents-medical-students/residents/fellowships/external-fellowships-and-awards> (2020, accessed 31 March 2020).

124. American Psychiatric Association. Awards, competitions, and leadership opportunities, <https://www.psychiatry.org/residents-medical-students/residents/awards-and-competitions> (2020, accessed 31 March 2020).
